# Liver-spleen axis, insulin-like growth factor-(IGF)-I axis and fat mass in overweight/obese females

**DOI:** 10.1186/1479-5876-9-136

**Published:** 2011-08-16

**Authors:** Silvia Savastano, Carolina Di Somma, Genoveffa Pizza, Annalba De Rosa, Valeria Nedi, Annalisa Rossi, Francesco Orio, Gaetano Lombardi, Annamaria Colao, Giovanni Tarantino

**Affiliations:** 1 Department of Molecular and Clinical Endocrinology and Oncology, Division of Endocrinology; Federico II University Medical School, Via S. Pansini 5-80131 Naples-Italy; 2 IRCCS SDN Foundation, Via Crispi, 8-80121 Naples-Italy; 3 Endocrinology, Parthenope University; Via Ammiraglio F. Acton 38-80133 Naples, Italy; 4 Department of Clinical and Experimental Medicine; Federico II University Medical School, Via S. Pansini 5-80131 Naples-Italy

## Abstract

**Background:**

Fat mass (FM) in overweight/obese subjects has a primary role in determining low-grade chronic inflammation and, in turn, insulin resistance (IR) and ectopic lipid storage within the liver. Obesity, aging, and FM influence the growth hormone/insulin-like growth factor (IGF)-I axis, and chronic inflammation might reduce IGF-I signaling. Altered IGF-I axis is frequently observed in patients with Hepatic steatosis (HS). We tested the hypothesis that FM, or spleen volume and C-reactive protein (CRP)--all indexes of chronic inflammation--could affect the IGF-I axis status in overweight/obese, independently of HS.

**Methods:**

The study population included 48 overweight/obese women (age 41 ± 13 years; BMI: 35.8 ± 5.8 kg/m^2^; range: 25.3-53.7), who underwent assessment of fasting plasma glucose and insulin, homeostasis model assessment of insulin resistance (HOMA), cholesterol and triglycerides, HDL-cholesterol, transaminases, high-sensitive CRP, uric acid, IGF-I, IGF binding protein (BP)-1, IGFBP-3, and IGF-I/IGFBP-3 ratio. Standard deviation score of IGF-I according to age (zSDS) were also calculated. FM was determined by bioelectrical impedance analysis. HS severity grading (score 0-4 according liver hyperechogenicity) and spleen longitudinal diameter (SLD) were evaluated by ultrasound.

**Results:**

Metabolic syndrome (MS) and HS were present in 33% and 85% of subjects, respectively. MS prevalence was 43% in subjects with increased SLD. IGF-I values, but not IGF-I zSDS, and IGF-I/IGFBP-3 ratio were significantly lower, while FM%, FPI, HOMA, ALT, CRP, were significantly higher in patients with severe HS than in those with mild HS. IGF-I zSDS (r = -0.42, r = -0.54, respectively; p < 0.05), and IGFBP-1 (r = -0.38, r = -0.42, respectively; p < 0.05) correlated negatively with HS severity and FM%. IGF-I/IGFBP-3 ratio correlated negatively with CRP, HS severity, and SLD (r = -0.30, r = -0.33, r = -0.43, respectively; p < 0.05). At multivariate analysis the best determinants of IGF-I were FM% (β = -0.49; p = 0.001) and IGFBP-1 (β = -0.32; p = 0.05), while SLD was in the IGF-I/IGFBP-3 ratio (β = -0.43; p = 0.004).

**Conclusions:**

The present study suggests that lower IGF-I status in our study population is associated with higher FM, SLD, CRP and more severe HS.

## Background

Adipose tissue produces a large number of inflammatory molecules responsible for low-grade chronic inflammation and insulin resistance (IR) [1]. In obese non-diabetic adults, the prevalence of nonalcoholic fatty liver disease (NAFLD) or, generally speaking, hepatic steatosis (HS), is high and is considered a further expression of metabolic syndrome (MS) [2]. Ultrasound (US) is widely used to detect HS [3] with high specificity, although it underestimates the prevalence of HS when there is < 20% fat [4]. Tsushima et al. first emphasized the role of the spleen in NAFLD patients [5]. It has been recently proposed that increased spleen volume--a stable index of chronic inflammation and activation of the immune system, and elevated concentrations of high sensitivity (hs)-CRP, both characterize young adult obese subjects with HS [6], just as high IL-6 levels coupled with larger spleen is suggestive of severe HS [7].

Up to 90% circulating insulin-like growth factor (IGF)-I, the main anabolic effector of Growth hormone (GH), originates in the liver, and hepatocytes represent also the largest source of IGF-binding protein (BP)-1 and IGFBP-3, the main IGF-I plasma carriers that regulate IGF-I bioavailability [8]. However, aging and a number of inflammatory cytokines are also known to affect IGF-I secretion from hepatocytes [9]. A number of clinical investigations have evaluated the interaction between HS [10], inflammation [11], and the IGF-I pathway; however, considering the effect of aging on IGF-I status and the role of IGF-I axis on body composition [12], no evidence of an association between HS, age-corrected IGF-I values, anthropometric data and spleen enlargement has been produced. Thus, we tested the hypothesis that the obesity-related low-grade chronic inflammation, evaluated by spleen volume and C-reactive protein (CRP), could affect the IGF-I axis status in overweight/obese, independently of HS.

## Patients and Methods

### Subjects

One hundred and thirteen overweight/obese women were consecutively selected to enter this study. They were referred to our Departments from October 1^st^, 2008 to July 31^st^, 2009 to participate in a weight loss program and/or to be evaluated as bariatric surgery candidates. In particular, female gender and age range were introduced as inclusion criteria to minimize the confounding effects of aging and sex-steroids on IGF-I metabolism [8]. Patients were on mild hypocaloric diet and reported exercising regularly 3 h/week. None of them had taken weight loss drugs or dietary supplements for at least three weeks before enrolment. The final population included 48 individuals--14 of whom in the post-menopausal status, mean age and BMI of 41 ± 13 years and 35.8 ± 5.8 Kkg/m^2 ^(range 25.3-53.7; 75 percentile 38.8 <; 95 5 CI 37.140.9).

### Exclusion criteria

i) Absence of T2D; parasitic infestations, microcythemia; chronic liver diseases of viral, alcoholic or autoimmune nature, or advanced NAFLD characterized by liver fibrosis; renal failure; cancer and acute viral, bacterial or fungal infection; the presence/absence of the above conditions was determined by complete medical examinations and/or laboratory investigations aimed at evaluating serum HCV-RNA, serum HBV-DNA; alcoholism at random, MCV, serum ferritin; serum ANA and AMA; AST/platelet ratio index; serum uric acid and creatinine; neoplastic markers; biological liquid culture; ii) absence of any other pituitary deficiency [13].

Out of 113 initial patients selected, we excluded eight patients who were older than 65 years; ten who were on metformin, nine on statins and/or clofibrate and seven on levothyroxine. In addition, 12 patients were excluded because they were being treated with low-doses of aspirin, two who were on hormone replacement therapy, fourteen who had joined previous weight loss programs and three who suffered from arthritis, bronchial asthma and chronic inflammatory bowel.

### Study design

This prospective study was conducted in accordance with the guidelines of the Declaration of Helsinki. The study was approved by the Ethics Committee of the Federico II University Medical School of Naples, (#231/05, February 20, 2006). All participants gave written consent. The primary outcome measures were the sonographic quantification of HS and spleen longitudinal diameter (SLD) at US, in addition to BMI, waist circumference, FM, IGF-I, IGFBP-1, IGFBP-3, IGF-I/IGFBP-3 ratio measurements. Secondary outcome measures were homeostasis model assessment of insulin resistance (HOMA), cholesterol and triglycerides, HDL-cholesterol, transaminases, CRP, and serum uric acid (UA).

### Laboratory data

All biochemical analyses including fasting plasma glucose (FPG), total cholesterol, HDL cholesterol, LDL cholesterol, triglycerides, transaminases, and uric acid were performed with a Roche Modular Analytics System in the Central Biochemistry Laboratory of our Institution. LDL and HDL cholesterol were determined by direct method (homogeneous enzymatic assay for the direct quantitative determination of LDL and HDL cholesterol). QC was performed with Bio-Rad's Quality Control Products., CRPwas measured using commercially available assays. T2D was defined as fasting blood glucose levels ≥ 126 mg/dL on two separate determinations, while Impaired Fasting Glucose (IFG) was defined as fasting blood glucose levels ≥ 110 < 126 mg/dL [14]. MS was diagnosed according to the revised Adults Treatment Panel III (2001), and three or more of the diagnostic criteria considered were: plasma glucose concentration of at least 100 mg/dL, waist circumference (WC) > 88 cm, serum HDL concentration < 50 mg/dL, blood pressure of at least 130/85 mmHg, and serum triglyceride concentrations of at least 150 mg/dL [15]. Fasting plasma insulin (FPI) was measured by a solid-phase chemiluminescent enzyme immunoassay using commercially available kits (Immulite 2000; Diagnostic Products Co, Los Angeles, CA, USA), the upper limit of the normal range being 15.6 μU/mL. HOMA was calculated according to Matthews et al [16]. As a stringent measure of IR, a value of HOMA > 2 was introduced [16]. Serum IGF-I levels were measured by IRMA after ethanol extraction using Diagnostic System Laboratories Inc. (Webster, Texas, USA). The sensitivity of the assay was 0.8 μg/L; the normal ranges in adults aged 20-40 and 41-60 years were 110-494 and 100-300 μg/l, respectively. The intra-assay CVs were 3.4, 3.0, and 1.5% for low, medium, and high points on the standard curve, respectively; inter-assay CVs were 8.2, 1.5, and 3.7% for low, medium, and high points on the standard curve, respectively. IGFBP-1 and IGFBP-3 levels were measured by ELISA (DSL Inc, Webster, TX). IGFBP-1 assay had a sensitivity of 0.25 ng/l; the intra and inter-assay coefficients of variation were 1.7-4.6% and 6.2-7.6%, respectively; the normal range for an adult female population in the same age range as our study population is 2670-5580 ng/ml. IGFBP-3 assay had a sensitivity of 0.04 ng/l; the intra and inter-assay coefficients of variation were 1.8-3.9% and 0.6-1.9%, respectively; the normal range for an adult female population in the same age range as our study population is 2670-5580 ng/ml. IGF-I/IGFBP-3 ratio was calculated as a indirect measure of free IGF-I. The values for the molecular mass of IGF-I and IGFBP-3 used for the calculation were 7.649 kDa and 28.5 kDa, respectively [17].

### Anthropometric evaluation

Obesity-related anthropometric measurements were made with the patients wearing only light underclothing and no shoes. Body weight was determined to the nearest 50 g using a calibrated balance beam scale. Body mass Index (BMI) was calculated as weight (kg) divided by height squared (m^2^) and used as an index of obesity. Subjects were classified as overweight or obese on the basis of BMI cut-off points of ≥ 25.0 and ≤ 29.9 kg/m^2^, respectively. WC was measured at the mid-point between the umbilicus and the xiphoid. In pre-menopausal women, the data were obtained during the early follicular phase, 5-7 days after spontaneous menses.

### Biompedance analysis

Body composition was determined by conventional bioelectrical impedance analysis and by bioelectrical impedance vector analysis with a single-frequency 50-kHz bioelectrical impedance analyzer (BIA 101 RJL, Akern Bioresearch, Florence, Italy), according to the standard tetrapolar technique, and employing the software provided by the manufacturer [18]. Patients were evaluated for FM% after an overnight fast and were asked to refrain from strenuous exercise and to maintain their usual intake of caffeinated beverages during the 3 days preceding the measurements.

### Ultrasound analyses

Sonographic measurements were performed by the same operator, blinded to patients' data, using a *Vivid *system (General Electric Healthcare Company, Milan, Italy). Briefly, SLD, the best single measurement well related to spleen size, was measured by postero-lateral scanning. Maximum and cranio-caudal lengths were measured and then averaged. A cut off for LSD was set at 103 mm [7]. The classification of "bright liver" or HS severity was based on the following scale of hyper-echogenity: 0 = absent, 1 = light, 2 = moderate, 3 = severe, pointing out the difference between the densities of the liver and the right kidney [19]. Technically, echo intensity can be influenced by many factors, particularly by gain intensity. To avoid confounders that could modify echo intensity and thus bias the comparisons, the mean brightness levels of both liver and right kidney cortex were obtained on the same longitudinal sonographic plane. The levels of brightness of liver and right kidney were calculated three times directly from the frozen images.

### Non-invasive liver fibrosis assessment

The aspartate aminotransferase (AST)/platelet ratio index was calculated as follows: AST level (U/L)/Upper normal limit for AST (35 U/L)/Platelet count (10/L) × 100.

### Statistical Analysis

Data were expressed as Mean ± SD. Since IGF-I is related to age, to analyze the relationships between IGF-I levels and the other variables, we calculated the standard deviation score (SDS) of IGF-I levels according to age (zSDS). To this aim, we calculated the mean and SD of IGF-I levels in adults (21-40 years) and middle-aged (41-65 years) women [20]. Differences in variables between groups according to HS classification were analyzed using ANOVA with the Bonferroni post-hoc test. Pearson's r or Spearman rho coefficients tests were used to analyze the association between variables when opportune. AST variable was log transformed. The presence of independent and significant associations between MS and SLD (< or > 103 mm) in the study groups was analyzed using multiple logistic regression, calculating the odds ratio (OR) and 95% confidence interval (CI). Using IGF-I, IGFBP-1, and IGF-I/IGFBP-3 ratio as dependent variables, three multiple linear regression analysis models were performed with the enter selection methods to evaluate the relative importance of HS score and FM% on IGF-I and IGFBP-1, respectively, and of CRP, HS score, transaminases, and SLD on IGF-I/IGFBP-3 ratio. To determine which variables contributed more or less to the regression equation, the standardized regression coefficient, or beta, and its ratio to the respective SE, i.e., the t-test, were calculated. To avoid multicollinearity, i.e., situations in which the predictors are correlated to each other to some degree, the variance inflation factor and tolerance were set at > 10 and < 0.1, respectively. P values < 0.05 were considered statistically significant. The concordance correlation coefficient (ρ_c_), which measures precision and accuracy, was adopted to evaluate the degree of intra-observer variation at US. Data were stored and analyzed using IBM SPSS Statistics 18.0 (SPSS Statistics, Chicago, IL, USA) and MedCalc^® ^package.

## Results

The concordance correlation coefficient to evaluate the degree of intra-operator variation at US for HS detection and spleen measurements was high (ρ_c _= 0.91). To rule out any interference of estrogens, data were analyzed grouping overweight/obese women according to menopausal status (table 1). Although there was a trend for higher FM% (p = 0.09) and systolic blood pressure (p = 0.06) among menopausal women, there were no significant differences in any of the variables between pre and postmenopausal subjects.

**Table 1:** Obesity-related anthropometric measurements and metabolic components in moderately-severely obese females, grouped according to menopausal status

	Study group	Pre-menopause	Post-menopause	Normal values/ Range	p values
Subjects	n.48	n. 34	n. 14		

BMI	35.5 ± 6.2	35.4 ± 6.8	35.6 ± 4.5	/	NS

Waist circumference	110.8 ± 15.9	110.1 ± 16.5	112.6 ± 14.6	< 88 cm	NS

Fat mass %	40.1 ± 7.2	39.2 ± 8.8	42.2 ± 4.7	16-30%	NS

FPG	92.4 ± 11.5	91.1 ± 11.4	95.8 ± 11.6	60-110 mg/dl	NS

FPI	14.4 ± 9.2	14.4 ± 9.0	14.1 ± 10.2	1-20 μU/ml	NS

HOMA	3.3 ± 2.2	3.3 ± 2.1	3.5 ± 2.8	≤ 2.5	NS

Total cholesterol	198.1 ± 33.2	192.6 ± 30.4	211.5 ± 37.3	≤ 190 mg/dl	NS

HDL cholesterol	52.3 ± 21.3	54.4 ± 24.7	50.3 ± 7.6	≥ 45 mg/dl	NS

Triglycerides	124.5.8 ± 72.2	121.6.8 ± 62.1	142.0 ± 81.9	≤ 150 mg/dl	NS

SBP	129.4 ± 17.8	126.3 ± 14.7	136.7 ± 22.4	≤ 120 mmHg	NS

DBP	84.1 ± 10.8	83.4 ± 10.2	89.3 ± 11.9	≤ 80 mmHg	NS

AST	23.6 ± 14	18.9 ± 3.8	24.3 ± 14.5	< 35 U/L	NS

ALT	32.5 ± 31.3	33.8 ± 32.6	23.3 ± 6.9	< 35 U/L	NS

Uric acid	4.5 ± 1.2	4.4 ± 1.0	4.6 ± 09	2.4-5.7 mg/dl	NS

CRP	2.9 ± 2.7	3.2 ± 2.5	2.4 ± 2.1	≤ 1.6 mg/dl	NS

SLD	112.9 ± 12.9	112.9 ± 12.9	109.5 ± 12.2	≤ 110 mm	NS

IGF-I	167.8 ± 80.2	175.5 ± 91.5	148.4 ± 36.5	100-494 μg/l	NS

IGF-I zSDS	-1.9 ± 1.7	-1.9 ± 2.0	-2.0 ± 1.0	/	NS

IGFBP-1	18.8 ± 14.5	19.4 ± 15.8	17.5 ± 12.6	/	NS

IGFBP-3	4445.4 ± 1331.1	4478.8 ± 1290.5	4369.3 ± 1467.2	2670-5580 ng/ml	NS

IGF-I/IGFBP3	0.027 ± 0. 01	0.027 ± 0. 01	0.028 ± 0.01	/	NS

AST/platelet index ratio	0.45 ± 4.7	0.44 ± 4.2	0.48 ± 5.6	< 0.76	NS

Hepatic steatosis	41	29	12	/	NS

Data are reported as mean ± SD. *FPG*, fasting plasma glucose; *FPI*, fasting plasma insulin; *HOMA*, homeostasis model assessment of insulin resistance; *SBP*, systolic blood pressure; *DBP*, diastolic blood pressure; *AST*, aspartate aminotransferase; *ALT*, alanine aminotranferase; *CRP*, C-reactive protein; *SLD*, spleen longitudinal diameter; *IGF-I*, Insulin-like growth factor-I; *IGF-I zSDS*, SDS of IGF-I levels according to age; *IGFBP-1*, IGF-binding protein-1; *IGFBP-3*, IGF-binding protein-3; *IGF-I/IGFBP-3*, IGF-I/IGFBP-3 ratio.

MS, HS, IFG were present in 33% (16), 85% (41), and 8% (4) of subjects, respectively. According to HS results, 7 subjects achieved a score of 0; 14 a score of 1 and 2; 13 a score of 3. The AST/platelet ratio index was higher, albeit not significantly, in HS scores 2-3. Table 2 shows the data obtained grouping subjects with HS scores of 0-1 and 2-3. IGF-I values, but not IGF-I zSDS, and IGF-I/IGFBP-3 ratio were significantly lower, while FM%, FPI, HOMA, alanine aminotransferase (ALT), CRP, were higher in patients with HS scores 2-3 than in those who had scored 0-1. According to cut off of 103 mm for SLD, MS prevalence was 43% in subjects with SLD > 103 mm, and 1% in subjects with SLD < 103 mm (χ^2 ^= 4.2; p = 0.04); thus, the likelihood of having MS was highest in the SLD > 103 mm subgroup (OR: 7.5; 95% CI 0.86 to 65.2).

**Table 2:** Obesity-related anthropometric measurements and metabolic components in moderately-severely obese females, grouped according to hepatic steatosis score at ultrasound (US) and in normal-weight controls matched for age and sex

	Hepatic steatosis score at US		p values
	**0-1 score** **(21/48)**	**2-3 grade** **(27/48)**	

Age(years)	40.6 ± 14.7	40.6 ± 12.3	NS

BMI	32.7 ± 5.8	37.4 ± 5.8	NS

Waist circumference(cm)	107.8 ± 17.3	113.0 ± 14.9	NS

Fat mass %	35.7 ± 8.0	43.6 ± 6.0	< 0.001

FPG (mg/dl)	92.8 ± 14.2	92.5 ± 8.4	NS

FPI (μU/ml)	10.7 ± 5.3	17.6 ± 10.7	0.021

HOMA	2.5 ± 1.5	4 ± 2.5	0.037

Total cholesterol (mg/dl)	197.0 ± 30.9	201.9 ± 33.3	NS

HDL- cholesterol (mg/dl)	58.2 ± 29	49.8 ± 9.3	NS

Triglycerides (mg/dl)	111.7 ± 60.8	137.8 ± 81	NS

SBP (mmHg)	129.5 ± 17.2	130.4 ± 18.0	NS

DBP (mmHg)	84.6 ± 8.7	84.5 ± 11.5	NS

AST (U/L)	21.6 ± 6.0	26.2 ± 18.5	NS

ALT (U/L)	25.2 ± 10.8	49.9 ± 21.6	0.045

Uric acid (mg/dl)	4.4 ± 1.0	4.5 ± 1.1	NS

CRP (mg/dl)	1.9 ± 2.1	3.6 ± 3.0	0.04

SLD (mm)	108.5 ± 10.5	124.2 ± 13.7	0.004

AST/platelet ratio index	0.44 ± 3.2	0.47 ± 2.5	NS

IGF-I (μg/l)	203.8 ± 94.8	138.0 ± 54.0	0.004

IGF-I zSDS	-1.2 ± 1.8	-2.5 ± 1.5	0.08

IGFBP-1	16.0 ± 3.7	12.8 ± 2.5	0.03

IGFBP-3 (ng/ml)	4526.8 ± 1321.8	4311.5 ± 1325.8	NS

IGF-I/IGFBP3 ratio	0.032 ± 0.01	0.023 ± 0.01	0.002

Data are reported as mean ± SD. *FPG*, fasting plasma glucose; *FPI*, fasting plasma insulin; *HOMA*, homeostasis model assessment of insulin resistance; *SBP*, systolic blood pressure; *DBP*, diastolic blood pressure; *AST*, aspartate aminotransferase; *ALT*, alanine aminotranferase; *CRP*, C-reactive protein; *SLD*, spleen longitudinal diameter; *IGF-I*, Insulin-like growth factor-I; *IGF-I zSDS*, SDS of IGF-I levels according to age; *IGFBP-1*, IGF-binding protein-1; *IGFBP-3*, IGF-binding protein-3; *IGF-I/IGFBP-3*, IGF-I/IGFBP-3 ratio.

Correlations between the study variables are reported in Table 3. As expected, BMI correlated positively with HOMA and HS severity, whereas transaminase levels correlated positively with HS severity; moreover, a positive correlation was also evident between BMI and SLD, and/or between transaminases and UA. FM% also correlated positively with HOMA and HS severity.

**Table 3:** Correlations between obesity-related anthropometric measurements and metabolic components in moderately-severely obese females

	BMI	*p *	*HOMA*	*p*	HS	*p *	ALT	*p*	AST	*p*	SLD	*p*
**BMI**	/		0.45	< 0.001	0.28	0.05	-0.38	0.01			0.46	0.001

**HOMA**	0.45	< 0.001	/				0.41	0.01			0.40	0.02

**HS**	0.28	0.05	0.34	0.04	/		0.30	0.05	0.31	0.05		

**CRP**	0.31	0.034			0.28	0.05	0.33	0.04			0.69	< 0.001

**Uric acid**							0.51	0.001	0.40	0.02	0.355	0.02

**FM%**	0.50	< 0.001	0.35	0.034	0.50	< 0.001	0.33	0.03				

*HOMA*, homeostasis model assessment of insulin resistance; *HS*, hepatic steatosis; *AST*, aspartate aminotransferase; *ALT*, alanine aminotransferase; *CRP*, C-reactive protein; *FM%*, Fat Mass percentage.

**Figure 1:**
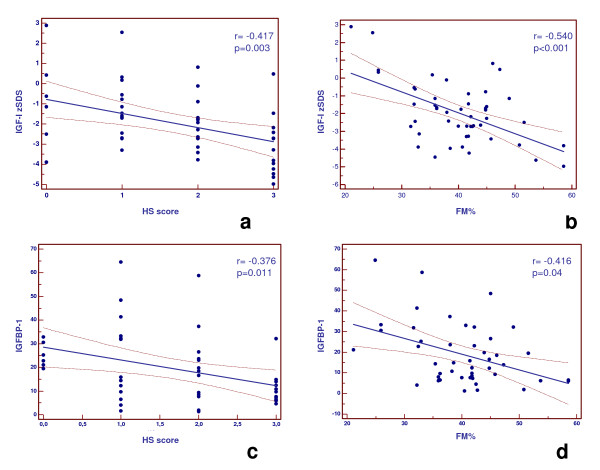
**Correlation between IGF-I zSDS (a and b) and IGFBP-1 (c and d), with HS score at ultrasound (US), and FM%**. *IGF-I zSDS*, standard deviation score (SDS) of insulin-like growth factor-I levels according to age; *IGFBP-1*, IGF-binding protein-1; *HS*, hepatic steatosis; *FM%*, percentage of fat mass. The SDS of IGF-I levels were calculated according to age (zSDS). The classification of "bright liver" or HS was based on the following scale of hyperechogenicity: 0 = absent, 1 = light, 2 = moderate, 3 = severe, pointing out the difference between the densities of the liver and the right kidney. FM was determined by conventional bioelectrical impedance analysis and by bioelectrical impedance vector analysis with a single-frequency 50-kHz bioelectrical impedance analyzer.

**Figure 2:**
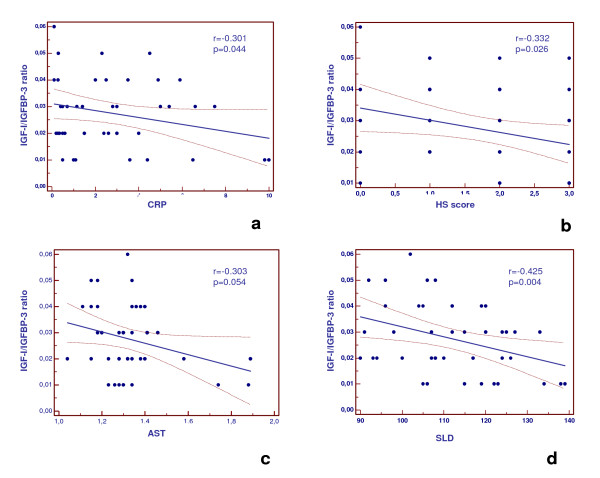
**Correlation between IGF-I/IGFBP-3 ratio with CRP (a), HS severity at ultrasound (US) (b), AST (c), and spleen longitudinal diameter (SLD) measured by postero-lateral scanning at US (d)**. *IGF-I*, insulin-like growth factor-I; *IGFBP-3*, IGF-binding protein-3; *HS*, hepatic steatosis, *SLD*, spleen longitudinal diameter. AST values were log transformed. SLD was measured by postero-lateral scanning. The maximum and cranio-caudal lengths were measured and then averaged.

IGF-I zSDS and IGFBP-1 correlated negatively with FM% and HS severity at US (Figure 1a, b, c, and 1d). Similarly, IGF-I/IGFBP-3 ratio correlated negatively with CRP, HS severity, and also SLD; *vice versa*, AST was not significantly associated with these variables (Figure 2a, b, d, and 2c). WC did not correlate with IGF-I levels (r = 0.12, p = 0.93).

At multivariate analysis the highest values of FM% well predicted the lowest levels of both IGF-I (β = -0.49; t = -3.67; p = 0.001) and IGFBP-1 (β = -0.32; t = -2.07; p = 0.05), whereas SLD was the best determinant of the IGF-I/IGFBP-3 ratio (β = -0.43; t = -3.04; p = 0.004), since a major spleen enlargement was associated with a lower IGF-I/IGFBP-3 ratio.

## Discussion

The results of this study underline that spleen enlargement, a parameter expressing low-grade chronic inflammatory status, was a major determinant of low IGF-I/IGFBP-3 ratio than HS *per se*. We also found a significant negative correlation between all the components of the IGF-I axis investigated and FM%, HOMA, or HS severity. However, FM%. was a better determinant of IGF-I and IGFBP-1 than HS *per se *in the same population. To the best of our knowledge, these associations are novel, and might contribute to the understanding of the involvement of the liver-spleen axis and FM in the pathogenesis of low IGF-I status in obesity.

Previous results evidenced the association of low IGF-I levels and IGF-I/IGFBP-3 ratio with different degrees of hyperechogenic liver pattern [10]. To this regard, the present study adds new information on this association as it shows the relevant role of SLD, and extends the investigation to other components of the IGF-I axis, such as IGFBP-1, an emerging marker of HS severity in clinical situations [20], as is the case of IR [21]. Furthermore, to minimize the confounding effects of age and gender, we calculated SDS of IGF-I values according to age and included only overweight/obese women. Alterations in the activity of the GH-IGF-I axis, as well as in inflammatory processes [22], seem to be related to aging [23] and obesity [24]. As a matter of fact, we found that the relationship between IGF-I and HS is likely to become less evident when IGF-I is corrected for age. Nevertheless, when the relationship between GH/IGF-I status and FM was evaluated in the setting of severe obesity, this association was independent of age [25].

In this context FM, SLD increase, HS grade, and the impairment of the IGF-I axis might represent different aspects of the same process, i.e., the chronic inflammation status, as an example of the maladaptation of obesity and obesity-related metabolic disorders.

We need to be aware of limitations in interpreting the results of this study. Firstly, the cross-sectional study design does not evidence a causal relationships between the study variables. Secondly, the data were obtained from a homogeneous and motivated group of women and, therefore, cannot be generalized beyond the cases studied, whereas the exclusion of patients with T2DM could have limited the adequate assessment of MS prevalence. Thirdly, FM was evaluated by bioimpedance analysis, which is useful for large-scale studies but is not interchangeable with DEXA and should be interpreted with caution, although recent evidence testifies in favor of its interchangeability in the obesity setting [26]. Fourthly, liver and spleen have been assessed by US parameters, which are operator-dependent; in this study liver histology was not performed for ethical reasons, and the use Fibroscan is not advisable in this type of subjects [27]. However, low AST/platelet ratio index, a useful and highly sensitive noninvasive marker of hepatic fibrosis in patients with NAFLD [28], helps us rule out liver fibrosis of moderate-severe entity in our population, and the validity of US has been verified by a recent meta-analysis [29]. In any case, these points need to be addressed again in a larger population-based sample, using also MRI abdominal imaging, or by measurement of other cytokines (mainly IL-6) to further support the hypothesis that the impairment of the IGF-I axis in obesity might represent different aspects of the chronic inflammation status more than HS *per se*.

In conclusion, the present study evidenced a clear inverse association of IGF-I status with FM, spleen enlargement, CRP and HS, adding new information on the complex relationships between impaired IGF-I status, HS, inflammation, and obesity.

## Abbreviations

*FM*: (fat mass); *IR*: (insulin resistance); *HS*: (hepatic steatosis); *GH*: (growth hormone); *IGF-I*: (insulin-like growth factor-1); *IGFBP*: (IGF binding protein); *BMI*: (body mass index); *HOM*A: (homeostasis model assessment of insulin resistance index); *SLD*: (spleen longitudinal diameter); *CRP*: (high-sensitive C-reactive protein); *MS*: (metabolic syndrome); *NAFLD*: (non-alcoholic fatty liver disease); *NASH*: (non-alcoholic steatohepatitis); *CVD*: (cardiovascular diseases); *T2D: *(type 2 diabetes); *IFG: *(Impaired Fasting Glucose).

## Competing interests

The authors declare that they have no competing interests.

## Authors' contributions

SS, AC, GT conceived and designed the study. SS, CDS, AC, GT coordinated the acquisition of the data and carried out the statistical analysis. GP, ADR, VN, AR, FO carried out the clinical investigations. GT performed ultrasonography. SS and GT drafted the manuscript. AC and GL revised the manuscript. All authors read and approved the final manuscript.
